# Polyhydroxybutyrate (PHB) Production Using an Arabinose-Inducible Expression System in Comparison With Cold Shock Inducible Expression System in *Escherichia coli*

**DOI:** 10.3389/fbioe.2021.661096

**Published:** 2021-05-03

**Authors:** Suchada Chanprateep Napathorn, Sirirat Visetkoop, Onruthai Pinyakong, Kenji Okano, Kohsuke Honda

**Affiliations:** ^1^Department of Microbiology, Faculty of Science, Chulalongkorn University, Pathum Wan, Thailand; ^2^Program in Biotechnology, Faculty of Science, Chulalongkorn University, Pathum Wan, Thailand; ^3^International Center for Biotechnology, Osaka University, Suita, Japan

**Keywords:** polyhydroxybutyrate, araBAD promoter, *Cupriavidus necator*, recombinant *E. coli*, pH stat feeding strategy

## Abstract

*Cupriavidus necator* strain A-04 has shown 16S rRNA gene identity to the well-known industrial strain *C. necator* H16. Nevertheless, the cell characteristics and polyhydroxyalkanoate (PHA) production ability of *C. necator* strain A-04 were different from those of *C. necator* H16. This study aimed to express PHA biosynthesis genes of *C. necator* strain A-04 in *Escherichia coli* via an arabinose-inducible expression system. In this study, the PHA biosynthesis operon of *C. necator* strain A-04, consisting of three genes encoding acetyl-CoA acetyltransferase (*phaA*_A–04_, 1182 bp, 40.6 kDa), acetoacetyl-CoA reductase (*phaB*_A–04_, 741 bp, 26.4 kDa) and PHB synthase Class I (*phaC*_A–04_, 1770 bp), was identified. Sequence analysis of the *phaA*_A–04_, *phaB*_A–04_, and phaC_A–04_ genes revealed that *phaC*_A–04_ was 99% similar to *phaC*_H16_ from *C. necator* H16. The difference in amino acid residue situated at position 122 of *phaC*_A–04_ was proline, whereas that of *C. necator* H16 was leucine. The intact *phaCAB*_A–04_ operon was cloned into the arabinose-inducible araBAD promoter and transformed into *E. coli* strains Top 10, JM109 and XL-1 blue. The results showed that optimal conditions obtained from shaken flask experiments yielded 6.1 ± 1.1 g/L cell dry mass (CDM), a PHB content of 93.3 ± 0.9% (w/w) and a productivity of 0.24 g/(L⋅h), whereas the wild-type *C. necator* strain A-04 accumulated 78% (w/w) PHB with a productivity of 0.09 g/(L⋅h). Finally, for the scaled-up studies, fed-batch cultivations by pH-stat control in a 5-L fermenter of *E. coli* strains XL1-Blue harboring pBAD/Thio-TOPO-*phaCAB*_A–04_ and pColdTF-*phaCAB*_A–04_ in MR or LB medium, leading to a PHB production of 31.4 ± 0.9 g/L at 54 h with a PHB content of 83.0 ± 3.8% (w/w), a CDM of 37.8 ± 1.2 g/L, a Y_*P/S*_ value of 0.39 g PHB/g glucose and a productivity of 0.6 g PHB/(L⋅h) using pColdTF-*phaCAB*_A–04_ in MR medium. In addition, PHB production was 29.0 ± 1.1 g/L with 60.2 ± 2.3% PHB content in the CDM of 53.1 ± 1.0 g/L, a Y_*P/S*_ value of 0.21 g PHB/g glucose and a productivity of 0.4 g PHB/(L⋅h) using pBAD/Thio-TOPO-*phaCAB*_A–04_ in LB medium. Thus, a relatively high PHB concentration and productivity were achieved, which demonstrated the possibility of industrial production of PHB.

## Introduction

Microplastic pollution continues to induce negative impacts that harm both animals and ecosystems. Nevertheless, the plastic manufacturing sector has moved to a circular economy that calls for upstream and downstream industry collaborations in chemical and mechanical recycling to create new resins offered with recycled and biobased content (Hahladakis et al., 2020). However, awareness among consumers regarding the efficient postuse of petrochemical plastics, the benefits of green material and government regulations that encourage people to adopt eco-friendly alternatives still promote the demand for bioplastics and biodegradable plastics. Recently, global bioplastic production capacity has been estimated to increase from 2.11 million tons in 2019 to approximately 2.43 million tons in 2024. Bioplastics can widely be used in a wide variety of markets and applications, from packaging, catering products, consumer electronics, automotive, agriculture, textiles and a number of other segments. Bioplastics consist of diverse materials and are primarily focused on renewable materials, including starch, cellulose, or bioethanol. Among them, polyhydroxyalkanoates (PHAs) and innovative biopolymers, such as biobased polypropylene, have received the highest attention and showed strong growth rates. Biodegradable plastics, which include PHAs, polylactic acid (PLA), starch blends and others, account for 55.5% (over 1 million tons) of the global bioplastic production capacities. The production of biodegradable plastics is expected to increase to 1,33 million in 2024, especially due to PHA’s significant growth rates (Bioplastics, 2020).

Nowadays, PHAs are one of the most preferable solutions to alleviate the conventional plastic pollution, especially for the preparation of PHAs microbeads that is required for the biodegradation of microbeads in marine environment. PHAs are a class of well-known microbial polyesters that possess unique advantages of thermoplastic, biocompatible and biodegradable properties. Based on their true biodegradable properties in various environments, PHAs are considered environmentally sustainable biodegradable plastics. However, PHAs have not yet become competitive and ecofriendly alternative materials to petrochemical plastics compared with semi-biosynthetic polymers, PLA and polybutylene succinate (PBS). The major problem is their high cost of production resulting from raw materials, solvent used in polymer extraction and purification, long cultivation time in the wild-type strain and small amount of obtained PHAs, even at its high PHA content from non-growth-associated PHA-producing strains. Recently, metabolic engineering approaches in which the PHA biosynthesis genes *phaCAB* and other related genes are cloned into *Escherichia coli* have been used to increase the efficiency of PHA production.

In our previous studies, we reported new isolated *Cupriavidus necator* strain A-04 that has shown the ability to produce PHAs [formerly reported as *Alcaligenes* sp. A-04 and *Ralstonia eutropha* A-04 (Chanchaichaovivat, 1992; Chanprateep et al., 2008)]. First, it has shown the ability to produce PHB from fructose and molasses. In 5 L fermentor, batch cultivation, the PHB produced was 7.21 g/L with PHB content of 82.12% (w/w) at 72 h when ammonium sulfate and fructose were 1.5 and 30.0 g/L, respectively. The amount of PHB produced from cane molasses was less than that obtained from fructose. The amount of PHB produced from molasses was 2.3 g/L with PHB content of 53.66% (w/w) at 72 h when 20% (w/v) of molasses was used as a carbon source and 0.1 g/L of ammonium sulfate was a nitrogen source (Phonprapai, 1994). It has also shown the ability to produce copolymer of poly(3-hydroxybutyrate-co-3-hydroxuvalerate) [P(3HB-*co*-3HV)], poly(3-hydroxybutyrate-*co*-4-hydroxybutyrate) (P(3HB-*co*-4HB)], and terpolymer of poly(3-hydroxybutrate-*co*-3-hydroxyvalerate-*co*-4-hydroxybutyrate) [P(3HB-*co*-3HV-*co*-4HB) with a wide range of monomer compositions from various organic acids (Surathikajon, 1994; Chanprateep and Kulpreecha, 2006; Chanprateep, 2010). Then, it was taxonomic characterization and it possessed 99.78% 16S RNA sequence similarity with *C. necator* H16, but it differed in PHA production ability based on PHB yield (Y_*P/S*_, g-PHB/g-carbon) from butyric, valeric, γ-hydroxybutyric acid, and fructose (Chanprateep et al., 2008). It could incorporate a high mole fraction of monomeric 4-hydroxybutyrate monomeric into the poly(3-hydroxybutyrate-*co*-4-hydroxybutyrate) [P(3HB-*co*-4HB)] copolymer under a C/N ratio of 20 and the biocompatibility of P(3HB-*co*-4HB) produced was also reported (Chanprateep et al., 2010). Recently, it has also shown the ability to grow on pineapple wastes hydrolysate that contains mixtures of sugars and tolerate levulinic acid and 5-hydroxymethyl furfural, and a detoxification step was not required prior to the conversion of cellulose hydrolysate to PHB (Sukruansuwan and Napathorn, 2018). Therefore, we aimed to study the PHA biosynthesis operon of *C. necator* strain A-04 and to realize the capability of the PHA biosynthesis operon of *C. necator* strain A-04 when it was heterologously expressed in recombinant *E. coli*. There have been a number of exciting developments in the field of metabolic regulation in recent years, but only a few reports are based on the use of arabinose-inducible expression systems for PHA production (Horng et al., 2010; Wu et al., 2016). The objective of this work was to analyze *phaCAB* genes from the isolated *C. necator* strain A-04 and clone these genes for expression in an arabinose-inducible araBAD promoter (pBAD/Thio-TOPO vector) that have not been reported for PHB production. Here, we investigated the optimal conditions; L-arabinose concentration, four different media, glucose concentration for *phaCAB*_A–04_ expression under pBAD/Thio-TOPO vector in shake flask cultivation. In fed-batch cultivation, *phaCAB*_A–04_ expression under araBAD promoter was compared with the cspA promoter (a cold- and isopropyl-β-D-thiogalactoside (IPTG)-inducible vector, pColdTF) (Boontip, 2020) using a pH stat fed-batch cultivation strategy. Finally, high cell density of cell dry mass (CDM) 51.2 ± 1.0 g/L with PHB 29.0 ± 1.1 g/L was obtained in LB medium.

## Materials and Methods

### Strains and Plasmids

All bacterial strains and plasmids used in this study are listed in Table 1. *C. necator* strain A-04, which is a soil-isolated bacterium possessing 99.84% 16S rRNA identity to *C. necator* H16, was used as a wild-type PHB-producing strain (Chanprateep et al., 2008). *E. coli* JM109 [F′*traD36 proA*+*B*+*lacI^q^(lacZ)*Δ*M15*/Δ(*lac-proAB*) *glnV44 e14- gyrA96 recA1 relA1 endA1 thi hsdR17*] (Promega Corporation, Madison, WI, United States) was used for general genetic manipulation and for the production of PHB. *E. coli* Top10 [F^–^ *mcr*A Δ(*mrr-hsd*RMS-*mcr*BC) Φ80 l*ac*ZΔ M15 Δ*lac*X74 *rec*A1 *ara*D139 Δ(ara-leu)7697 *gal*U *gal*K *rps*L (Str^*R*^) *end*A1 *nup*G (Invitrogen, Carlsbad, CA, United States) was used for PHB production. *E. coli* XL1 Blue [*recA1 endA1 gyrA96 thi-1 hsdR17 supE44 relA1 lac* [F′ *proAB lacI*^*q*^*Z*Δ*M15* Tn*10* (Tet^*r*^)] (Promega Corporation, Madison, WI, United States) was used for PHB production in a 5 L fermenter. The pCR4-TOPO vector (Invitrogen, Carlsbad, CA, United States) was used for cloning the *phaCAB* and *phaC* genes. The pBAD/Thio-TOPO vector (Invitrogen, Carlsbad, CA, United States) was used as a final vector for the *phaCAB* expression plasmid. *C. necator* strain A-04 was cultivated in pre-culture medium consisting of 10 g/L yeast extract, 10 g/L polypeptone, 5 g/L beef extract, and 5 g/L (NH_4_)_2_SO_4_ at 30°C at 200 rpm for 24 h. *E. coli* JM109 and *E. coli* Top10 were cultivated at 37°C in Luria-Bertani (LB) medium containing 10 g/L casein peptone, 10 g/L sodium chloride and 5 g/L yeast extract. The antibiotic ampicillin (100 μg/mL) or carbenicillin (50 μg/mL) was added to the medium to maintain the stability of the plasmids.

**TABLE 1:** Bacterial strains and plasmids used in this study.

**Strains/plasmids**	**Relevant description**	**Reference/source**
**Strain**		
*Cupriavidus necator* strain A-04	Wild Type	Chanprateep et al., 2008
*Escherichia coli* JM109	F′*traD36 proA*+*B*+*lacI^q^(lacZ)*Δ*M15*/Δ*(lac-proAB) glnV44 e14- gyrA96 recA1 relA1 endA1 thi hsdR17*	Promega Corporation, Madison, WI, United States
*Escherichia coli* TOP10	F^–^ *mcr*A Δ(*mrr-hsd*RMS-*mcr*BC) Φ80 l*ac*ZΔ M15 Δ*lac*X74 *rec*A1 *ara*D139 Δ(ara-leu)7697 *gal*U *gal*K *rps*L (Str^*R*^) *end*A1 *nup*G	Invitrogen, Carlsbad, CA, United States
*Escherichia coli* XL1-Blue	*recA1 endA1 gyrA96 thi-1 hsdR17 supE44 relA1 lac* [F′ *proAB lacI*^*q*^*Z*?*M15* Tn*10* (Tet^*r*^)	Promega Corporation, Madison, WI, United States
**Plasmids**		
pGEM-T	Cloning vector, Amp^*r*^	Promega Corporation, Madison, WI, United States
pBAD/Thio-TOPO	Amp^*r*^, *araC* gene, *araBAD* promoter	Invitrogen, Carlsbad, CA, United States
pBAD/Thio-TOPO-*phaCAB*_A–04_	pBAD/Thio-TOPO derivative, carrying C-terminal 6HIS- and N-terminal thioredoxin fused *phaCAB* from *C. necator* strain A-04	This study
pBAD/Thio-TOPO-*phaC*_A–04_	pBAD/Thio-TOPO derivative; *araBAD* promoter, *phaC* from *C. necator* strain A-04	This study
pColdTF-*phaCAB*_A–04_	pColdTF derivative, carrying N-terminal 6His-fused *phaCAB* from *C. necator* strain A-04	Boontip, 2020

### Amplification of PHA Biosynthesis Genes From *C. necator* Strain A-04

Genomic DNA of *C. necator* strain A-04 was extracted by standard procedures (Sambrook and Russell, 2001). A partial fragment of the *phaC* gene from the genomic DNA of *C. necator* A-04 was amplified using two degenerate primers (F): 5′-TAYATHYTNGAYYTNCARCCNT-3′ and (R): 5′-CGYAGTHYCACAYYAGGTCG-3′, which were based on highly conserved regions of the *phaC* genes of *Comamonas acidovorans* (accession no. AB009273), *Alcaligenes* sp. strain SH-69 (accession no. U78047), *Alcaligenes eutrophus* (accession no. J05003), *Aeromonas caviae* (accession no. D88825) *Rhodococcus rubber* (accession no. X66407), *Chromatium vinosum* (accession no. 01112), and *C. necator* H16 (accession no. AM260479.1) (Sudesh et al., 1998). Next, the partial sequences (469 bp) of *phaC* were detected with degenerated PCR primers, and the subsequent sequences were obtained using the GenomeWalker^TM^ Universal Kit (Clontech Laboratories Inc., United States). In addition, two target-specific primers (forward primer (F0): 5′-TACATCCTGGACCTGCAGCC-3′ and reverse primer (R4): 5′-CGTAGTTCCACACCAGGTCG-3′) were designed based on the determined sequences. A GenomeWalker^TM^ Universal Kit was completed according to the manufacturer’s protocols. The amplified full-length genes of the entire *pha* locus containing the *phaC*_A–04_, *phaA*_A–04_, and *phaB*_A–04_ genes of *C. necator* strain A-04 were cloned into a pGEM-T easy vector (Promega, Madison, WI, United States) followed by transformation of the vectors into *E. coli* JM109 prior to DNA sequencing. DNA sequencing was performed at the Macrogen service center (Macrogen Inc., Seoul, Korea). The nucleotide sequences of *phaC*_A–04_, *phaA*_A–04_, and *phaB*_A–04_ reported here were deposited in the GenBank online database under accession numbers FJ897463.1, FJ897461.1, and FJ897462.1, respectively. In addition, the nucleotide sequences of the NAD-dependent 4-hydroxybutyrate dehydrogenase gene of *C. necator* strain A-04 were deposited in the GenBank online database under accession number MH243762.1.

### Construction of the Plasmids and Cloning of the *phaCAB* and *phaC* Genes

Specific primers for the amplification of *phaCAB* were designed according to the sequences obtained. The oligonucleotide primers for the amplification of the *phaCAB* gene fragment were (F-phaCAB): 5′-ATGGCGACCGGCAAAG-3′ (forward primer) and (R-phaCAB): 5′- TCAGCCCATATGCAGGCC-3′ (reverse primer). The oligonucleotide primers for the amplification of the *phaC* gene fragment were (F-phaCAB): 5′-ATGGCGACCGGCAAAG-3′ (forward primer) and (R-phaC): 5′-TCATGCCTTGGCTTTGACG-3′ (reverse primer). PCR using the respective primers for the amplification of *phaCAB* and *phaC* genes from the genomic DNA of *C. necator* strain A-04 was carried out to obtain the ligated DNA fragments using Ex Taq^TM^ DNA Polymerase (TaKaRa Bio Inc., Otsu, Japan) for ligation. The fragment was ligated to pBAD/Thio-TOPO^®^ (Invitrogen, Carlsbad, CA, United States) according to the manufacturer’s protocol. A new plasmid named pBAD/Thio-TOPO-*phaCAB*_A–04_ with the entire *phaCAB*_A–04_ operon and pBAD/Thio-TOPO-*phaC*_A–04_ with *phaC*_A–04_ were transformed using a heat shock method (Sambrook and Russell, 2001) into *E. coli* JM109 and *E. coli* TOP10. The colonies were screened on LB agar containing carbenicillin (50 μg/mL), and the inserts were confirmed through PCR and restriction enzyme digestion.

### Expression of pBAD/Thio-TOPO-*phaCAB*_A–04_ in *E. coli* JM109 and *E. coli* TOP10 and Comparison of Their PHB Production From Glucose

Shaken flask experiments were performed in 500-mL Erlenmeyer flasks containing 100 mL of medium. *E. coli* JM109 and *E. coli* TOP10 transformants containing pBAD/Thio-TOPO-*phaCAB*_A–04_ or pBAD/Thio-TOPO-*phaC*_A–04_ plasmid were grown in LB medium containing carbenicillin 50 μg/mL at 37°C and 200 rpm for 16–18 h. Subsequently, cells were harvested by centrifugation, washed to remove the medium and resuspended in 5 mL of 0.85% sodium chloride solution. The cells were separately inoculated into four different media: LB (10 g/L casein peptone, 10 g/L sodium chloride and 5 g/L yeast extract), superbroth (SB) (32 g/L tryptone, 5 g/L sodium chloride and 20 g/L yeast extract), 2YT (16 g/L tryptone, 5 g/L sodium chloride and 10 g/L yeast extract), and terrific broth (TB) (12 g/L tryptone, 4 mL glycerol and 24 g/L yeast extract) containing L-arabinose 0.2% (w/v), glucose 20 g/L and carbenicillin 50 μg/mL to evaluate suitable media for PHB production. Next, five concentrations of L-arabinose, 0.002, 0.02, 0.2, 0.5, and 2% (w/v), were separately added when an optical density at a wavelength of 600 nm (OD_600_) of 0.5–0.6 was obtained to induce the arabinose-inducible araBAD promoter for *phaCAB*_*A04*_ or *phaC*_*A04*_ expression. Finally, the inoculum size was investigated by means of a colony forming unit (CFU) varying from 4.5 × 10^8^, 3.2 × 10^9^, and 1.5 × 10^10^ CFU in LB medium containing L-arabinose 1.0% (w/v), glucose 20 g/L and carbenicillin 50 μg/mL. Culture samples were taken at 12 h intervals for 96 h.

### PHA Granule Observation by Transmission Electron Microscopy

Polyhydroxyalkanoate production was confirmed by comparative staining using the modified Sudan Black B staining method (López-Cortés et al., 2008). Cell morphology and PHA granules were also observed by transmission electron microscopy (TEM) at Scientific and Technological Research Equipment Centre Chulalongkorn University, Bangkok, Thailand. For TEM, diluted culture from production medium was fixed in 2% (v/v) glutaraldehyde containing 2% (v/v) paraformaldehyde in 0.1 M phosphate buffer (pH 7.2) and was postfixed in 1% (w/v) osmium tetroxide. The cells were dehydrated in an ascending series of ethanol concentrations from 35 to 100% and embedded in Spurr resin (EMS, PA, United States). Thin sections were prepared with an LKB 2088 Ultratome V (Surrey, United Kingdom), stained with 2% (w/v) uranyl acetate and 2% (w/v) lead citrate and examined with TEM (JEM 2100, JEOL Ltd., Tokyo, Japan) at an accelerating voltage of 80 kV (Muangwong et al., 2016).

### Conditions for PHB Production in a 5-L Fermenter

In this study, competent *E. coli* XL1 blue cells were transformed with pBAD/Thio-TOPO-*phaCAB*_A–04_ and pColdTF-*phaCAB*_A–04_. Seed cultures were prepared in LB medium containing 50 μg/mL carbenicillin or 100 μg/L ampicillin and 20 g/L glucose at 30°C and 200 rpm for 10 h at 200 rpm. For fed-batch culture, LB medium supplemented with 20 g/L glucose and MR medium supplemented with 20 g/L glucose and 10 mg/L thiamine were compared. MR medium, pH 7.0, consisted of the following: 13.5 g/L KH_2_PO_4_, 4.0 g/L (NH_4_)_2_HPO_4_, 1.4 g/L MgSO_4_⋅7H_2_O, 1.7 g/L citric acid and 10 mL/L trace metal solution. The trace metal solution contained the following (in 5 M HCl): 10.0 FeSO_4_⋅7H_2_O, 2.0 CaCl_2_, 2.2 ZnSO_4_⋅7H_2_O, 0.5 MnSO_4_⋅4H_2_O, 1.0 CuSO_4_⋅5H_2_O, 0.1 (NH_4_)_6_Mo_7_O_24_⋅4H_2_O, 0.02 Na_2_B_4_O_7_⋅10H_2_O (Kahar et al., 2005). The seed culture at 5% (v/v) was inoculated into a 5-L bioreactor (MDL500, B.E. Marubishi Co., Ltd., Tokyo, Japan) containing an initial volume of 2 L of LB medium supplemented with 50 μg/mL carbenicillin or 100 μg/L ampicillin and 20 g/L glucose in comparison with MR medium supplemented with 10 mg/L thiamine, 50 μg/mL carbenicillin or 100 μg/L ampicillin and 20 g/L glucose. Fed-batch cultures were carried out at 30°C, and the pH was maintained at 7.0 by automatic addition of 28% (v/v) ammonia hydroxide solution. The dissolved oxygen was monitored and controlled at above 10% air saturation by varying agitation and aeration. The feeding solution used for the fed-batch cultivation contained the following: 700 g/L glucose, 15 g/L MgSO_4_⋅7H_2_O and 0.25 g/L thiamine (Kahar et al., 2005). A pH stat feeding strategy using a pH electrode (Broadley-James Corporation, Irvine, CA, United States) based on increasing pH due to glucose depletion was used in this study. When the pH was greater than 7.0, an appropriate volume of feeding solution was fed to supply glucose to the culture medium. The fermenter was coupled to a process control system (Bio Process Controller, MDIAC-S5, B.E. Marubishi Co., Ltd., Tokyo, Japan). The agitation speed and the air flow rate were 500 rpm and 1 mL/min, respectively. After an OD_600_ of 0.5 was obtained, isopropyl-β-D-thiogalactopyranoside (IPTG) was added to the culture at a final concentration of 0.5 mM. Culture samples were collected at 6 h intervals for 72 h. For pColdTF-*phaCAB*_A–04_, after an OD_600_ of 0.5 was obtained, the cultivation temperature was reduced from 37 to 15°C for 30 min. Next, IPTG was added to the culture at a final concentration of 0.5 mM. After IPTG addition, the cultivation temperature was shifted from 15 to 37°C and maintained at 37°C for 72 h (Boontip, 2020). Culture samples were collected at 6 h intervals for 48 h.

### Analytical Methods

Cell growth was monitored by the CDM, which was determined by filtering 5 mL of the culture broth through pre-weighed cellulose nitrate membrane filters (pore size 0.22 μm; Sartorius, Göttingen, Germany). The filters were dried at 80°C for 2 days and stored in desiccators. The net biomass was defined as the residual cell mass (RCM), which was calculated by subtracting the amount of PHB from the CDM. The PHB in dried cells was methyl-esterified using a mixture of chloroform and 3% (v/v) methanol-sulfuric acid (1:1 v/v) (Braunegg et al., 1978). The resulting monomeric methyl esters were quantified by a gas chromatograph (model CP3800, Varian Inc., Walnut Creek, CA, United States) using a Carbowax-PEG capillary column (0.25-μm df, 0.25-mm ID, 30 m length, Agilent Technologies, Inc., Santa Clara, CA, United States). The internal standard was benzoic acid, and the external standard was PHB (Sigma-Aldrich Corp., St. Louis, MO, United States). The total reducing sugar concentration was determined using a 3,5-dinitrosalicylic acid (DNS) assay (Miller, 1959). The NH_4_^+^ concentration in the culture medium was determined using a colorimetric assay (Kemper, 1974).

### PHA Extraction and Purification

Harvested cells were dried and extracted with 1,3-dioxolane, and PHB was recovered from 1,3-dioxolane by precipitation in water. The precipitation step was repeated three times (Yabueng and Napathorn, 2018).

### Chemical Structure Analysis

^1^H and ^13^C NMR spectra of PHA samples were recorded on a Varian Inova 500 MHz instrument. The chemical shifts are reported in parts per million (ppm) relative to chloroform as an internal reference. Spectra were recorded using 5% (w/w) polymer solutions in CDCl3 with the following parameters: 25°C, pulse of 90 degrees, width of 8003.2 Hz, 5.0 s relaxation delay, and 0.2 Hz line broadening. Two-dimensional ^1^H-correlation spectroscopy (2D-^1^H-COSY) was performed in combination with ^1^H NMR on a Bruker AM 400 MHz FT-NMR (Bruker BioSpin Corporation, Woodland, TX, United States).

### Thermal Analysis by Differential Scanning Calorimetry (DSC)

A 10-mg sample of PHB was encapsulated in an aluminum sample vessel and placed in the sample holding chamber of the differential scanning calorimetry (DSC) apparatus (DSC7, PerkinElmer, Inc., Waltham, MA, United States). STARe software (version SW 10.00; Mettler-Toledo International Inc., Columbus, OH, United States) was used to operate the DSC apparatus at the Petroleum and Petrochemical College, Chulalongkorn University. The previous thermal history of the sample was removed before the thermal analysis by heating the sample from ambient temperature to 180°C at 10°C/min. Next, the sample was maintained at 180°C for 5 min before cooling at 10°C/min to −50°C. The sample was then thermally cycled at 10°C/min to 180°C. The melting peak temperature, denoted by T*_*m*_*, was given by the intersection of the tangent with the furthest point of an endothermic peak and the extrapolated sample baseline. The glass transition temperature, denoted by T*_*g*_*, could be estimated by extrapolating the midpoint of the heat capacity difference between glassy and viscous states after heating of the quenched sample.

### Data Analysis

All the data presented in this manuscript are representative of the results of three independent experiments and are expressed as the mean values ± standard deviations (SDs). Analysis of variance (one-way ANOVA) followed by Duncan’s test for testing differences among means was conducted using SPSS version 22 (IBM Corp., Armonk, NY, United States). Differences were considered significant at *P* < 0.05.

## Results

### Cloning of *phaCAB*_A–04_

**FIGURE 1:**
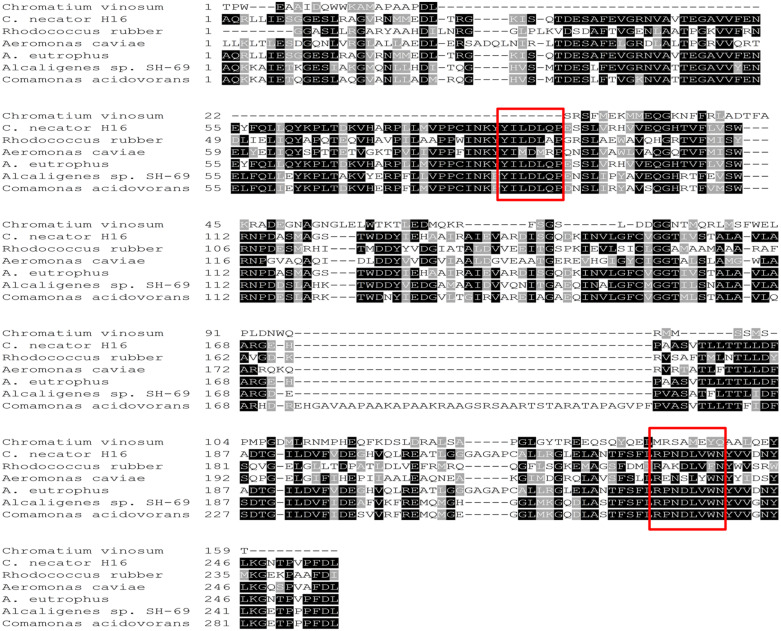
Alignment of the deduced amino acid sequence of the PHA synthase from *Comamonas acidovorans* (accession no. AB009273), *Alcaligenes* sp. strain SH-69 (accession no. U78047), *A. eutrophus* (accession no. J05003), *Aeromonas caviae* (accession no. D88825), *Rhodococcus rubber* (accession no. X66407), *Chromatium vinosum* (accession no. 01112), and *C. necator* H16 (accession no. AM260479.1) (Sudesh et al., 1998). Amino acids that are identical between all the PHA synthases are identified by black boxes, whereas conserved and similar amino acids are highlighted in light gray. The red box means the selected conserved sequence regions that were used for designing degenerate primers.

Based on our study, the element composition of biomass represented by CH_*a*_O_*b*_N_*c*_ for *C. necator* H16 was CH_1.77_O_0.50_N_0.24_ with ash content of 5.31% (w/w) and C/N ratio based on biomass element composition was 4.09 whereas those for *C. necator* strain A-04 was CH_1.66_O_0.45_N_0.16_ with ash content of 2.865% (w/w) and C/N ratio of 6.37. For identification of the PHA biosynthesis genes of *C. necator* strain A-04, a partial fragment of the *phaC*_A–04_ gene (469 bp) was amplified from the genomic DNA using degenerate primers based on the highly conserved regions of *phaC* genes of *C. acidovorans* (accession no. AB009273), *Alcaligenes* sp. strain SH-69 (accession no. U78047), *A. eutrophus* (accession no. J05003), *A. caviae* (accession no. D88825), *R. rubber* (accession no. X66407), *C. vinosum* (accession no. 01112), and *C. necator* H16 (accession no. AM260479.1), as shown in Figure 1. In addition, after optimizing PCR conditions, it was found that the partial fragment of the *phaC*_A–04_ gene (469 bp) and *phaC*_H16_ gene (as a positive control, 460 bp) could be amplified from the genomic DNA using different MgCl_2_ concentration where *C. necator* H16 required higher MgCl_2_ concentration than that of *C. necator* strain A-04. Although, there seems to be a low opportunity to obtain new results contributing to the existing knowledge, it is very challenging to us to express PHA biosynthesis genes of *C. necator* strain A-04 in recombinant *E. coli* based on an academic point of view.

**FIGURE 2:**
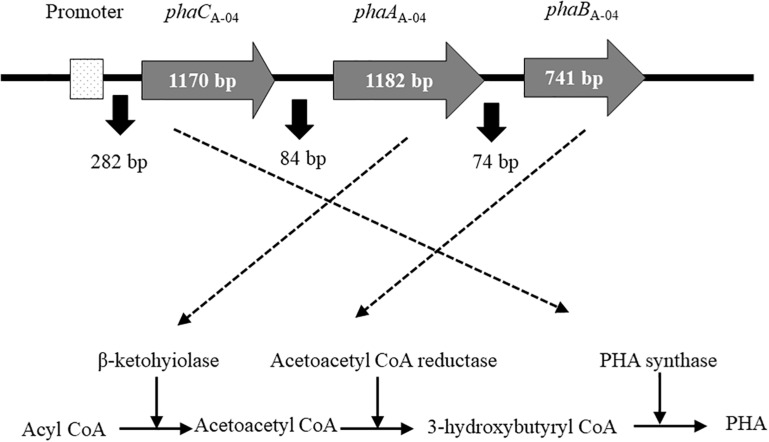
Diagram of the molecular organization of *phaCAB*_A–04_ biosynthesis genes from *C. necator* strain A-04. PHA synthase (*phaC*_A–04_), acetyl-CoA acetyltransferase (*phaA*_A–04_), acetoacetyl-CoA reductase (*phaB*_A–04_) genes and promoter (P_A–04_).

Next, the entire sequence of *phaCAB*_A–04_ was obtained using a gene walk kit by digesting genomic DNA with SmaI. The resulting 1.7 kb PCR product was obtained when the outer forward primer (F3: 5′-TCACGCTGCTGACCACGCTGCTGGACTTTG-3′) was used with outer adaptor primer 1 (AP1: 5′-GTAATA CGACTCACTATAGGGC-3′), whereas a PCR product as large as 4.0 kb was obtained when the nested forward primer (F4: 5′-TTGAGCTGGCCAATACCTTCTCGTTCTTGC-3′) was used with nested adaptor primer 2 (AP2: 5′-ACTATAGGGC ACGCGTGGT-3′). Analysis of the entire nucleotide sequence of the PCR product revealed that *phaA*_A–04_ and *phaB*_A–04_ were located downstream of the *phaC*_A–04_ gene, and the molecular organization of the *phaCAB*_A–04_ operon from *C. necator* strain A-04 is shown in Figure 2. The PCR product of the expected size (4.0 kb) was recovered, purified, and cloned into the pBAD/Thio-TOPO vector. They were transformed into *E. coli* TOP10, JM109 and XL-1 Blue as the expression host strains. Twenty of five-hundred transformants were screened for PHA production by Nile blue A staining of the colonies and investigated the light intensity under blue light (470 nm) (BluPAD dual LED blue/white light transilluminator, Bio-helix, Taiwan) using Image-J program. Five clones that showed either similar or higher levels of fluorescence intensity that increased with the increase in PHA content of PHA than that of *C. necator* A-04 were selected and further analyzed to ensure the correctness of sequences. There have been relatively few reports on the use of arabinose promoter for PHB production in recombinant *E. coli* (Horng et al., 2010; Wu et al., 2016). In this study, an arabinose-inducible araBAD promoter (pBAD/Thio-TOPO vector) that has not been reported for PHB production was used to produce PHB in *E. coli* TOP10, JM100 and XL-1 Blue. For a comparative study, the expression plasmid pColdTF (cspA promoter, cold shock and IPTG inducible) harboring *phaCAB*_A–04_ genes was transformed into *E. coli* XL-1 Blue.

### Selection of Growth Medium

**FIGURE 3:**
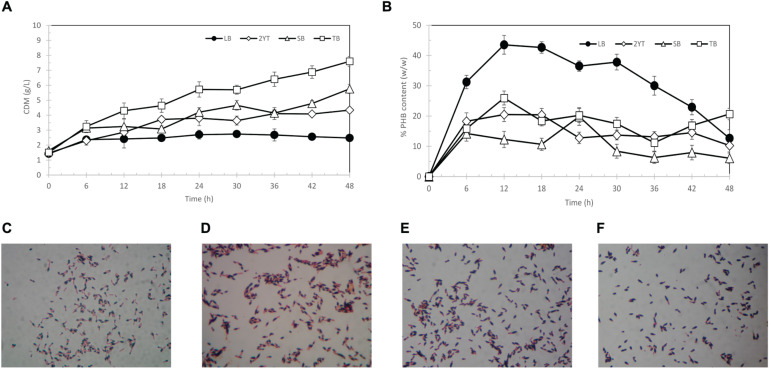
Time courses of CDM (g/L) **(A)** % PHB content (w/w) **(B)** and Sudan black B-stained PHB granules (black section) in pBAD/Thio-TOPO-*phaCAB*_A–04_ expressing *E. coli* TOP10 grown in LB medium at 6 h **(C)**, 12 h **(D)**, 18 h **(E)**, and 24 h **(F)**.

The choice of growth media has a significant influence on cell growth and PHB production. Thus, selection of suitable growth medium and conditions for PHB production in *E. coli* TOP10 was carried out by comparison of kinetic parameters. The results are shown in Figure 3. Terrific broth (TB) medium provided better growth conditions than the other three types of media. The specific growth rate of *E. coli* TOP10 harboring pBAD/Thio-TOPO-*phaCAB*_A–04_ in TB medium was 0.035 (1/h), followed by SB medium (0.027 (1/h)), 2YT (0.024 (1/h)), and LB medium (0.007 (1/h)). However, the LB medium appeared to promote PHB production in *E. coli* TOP10, resulting in a specific production rate of 0.048 g-PHB/g-CDW/h, whereas the specific production rate in TB medium was 0.012 g-PHB/g-CDW/h. TB medium contains the highest amount of yeast extract with additional glycerol, which makes it suitable for the long-term growth of cells but does not promote PHB production. The LB medium contained the lowest amount of yeast extract and tryptone with a slightly higher amount of sodium chloride and was found to promote PHB production better than TB, 2YT, and SB media. Figures 3C–F show time courses of PHB granules accumulated in *E. coli* TOP10 grown in LB medium at 6, 12, 18, and 24 h visualized by Sudan Black B staining. Finally, LB medium was chosen for further optimization (Nambu et al., 2020).

### Optimal Conditions for *phaCAB*_A–04_ Genes Expression

To optimize PHB production in recombinant *E. coli*, many variables need to be systematically investigated. In this study, arabinose concentration, length of induction, inoculum size and glucose concentration were investigated in detail.

**FIGURE 4:**
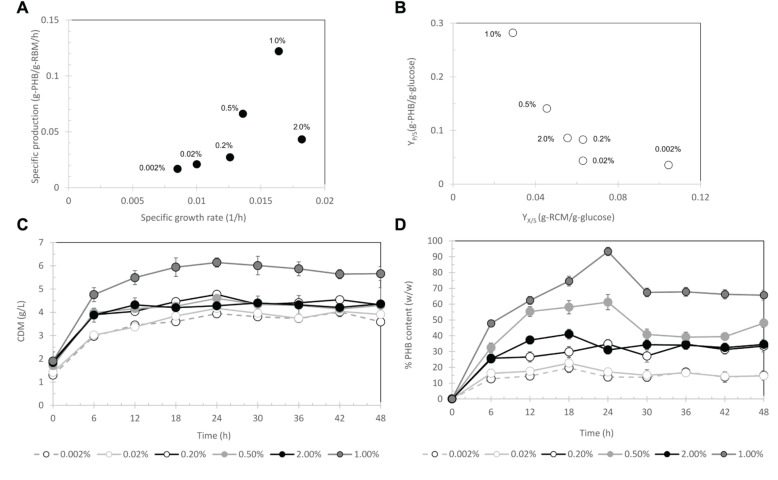
Effect of L-arabinose concentrations of 0.002, 0.02, 0.2, 0.5, and 2% (w/v) on the relationship between the specific growth rate and specific production rate **(A)** and the relationship between Y_*X/S*_ and Y_*P/S*_ **(B)** time course of CDM (g/L) **(C)** and % PHB content (w/w) **(D)**.

**FIGURE 5:**
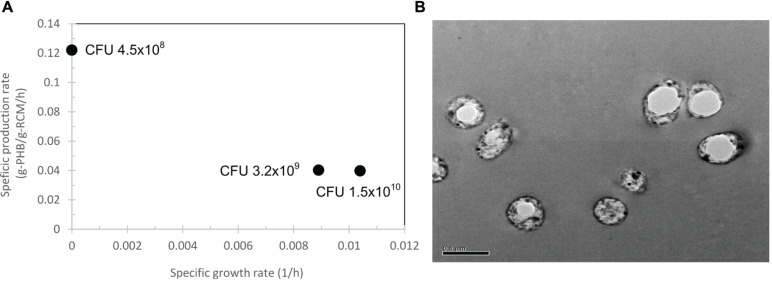
Effect of inoculum size from 4.5 × 10^8^, 3.2 × 10^9^, and 1.5 × 10^10^ CFU/mL on the specific growth rate and specific production rate **(A)** and transmission electron micrograph of ultrathin sections of pBAD/Thio-TOPO-*phaCAB*_A–04_ expressing *E. coli* TOP10 grown in LB medium. **(B)** Typical short rod cells containing one PHB granule (white fractions). Bar 0.5 μm.

First, the arabinose concentration was varied from 0.002, 0.02, 0.2, 0.5, and 2% (w/v) for pBAD/Thio-TOPO-*phaCAB*_A–04_-expressing *E. coli* TOP10. Figure 4A represents the effect of arabinose concentration on the relationship between the specific growth rate and specific production rate. The specific PHB production rate increased proportionally with increasing L-arabinose concentration from 0.002 to 1% (w/v), and then the specific PHB production rate decreased at an L-arabinose concentration of 2.0 (w/v). It was clearly observed that 1.0% (w/v) L-arabinose gave the highest amount of PHB at 5.6 ± 0.4 g/L (Figure 4C) with a PHB content of 93.3 ± 0.9% (w/w) at 24 h after induction (Figure 4D). The effect of arabinose concentration on Y_*P/S*_ (g-PHB/g-glucose) and Y_*X/S*_ (g-RCM/g-glucose) was shown in Figure 4B and it was clearly found that 1% (w/v) L-arabinose provided the highest Y_*P/S*_ of 0.28 g-PHB/g-glucose. Next, the inoculum size was varied from 4.5 × 10^8^, 3.2 × 10^9^, and 1.5 × 10^10^ CFU/mL. Figure 5A demonstrates the effect of inoculum size on the specific growth rate and specific production rate. In this study, using a low inoculum size of 4.5 × 10^8^ CFU/mL gave the highest specific production rate compared with those obtained from a high cell concentration of pre-culture. For the expression of pBAD/Thio-TOPO-*phaC*_A–04_-expressing *E. coli* TOP10 using the above optimal conditions, PHB production was not observed. Next, the comparison of pBAD/Thio-TOPO-*phaCAB*_A–04_ was compared between *E. coli* TOP10 and JM109. The optimal conditions of arabinose concentration, length of induction and inoculum size for pBAD/Thio-TOPO-*phaCAB*_A–04_-expressing *E. coli* JM109 were similar but they gave lower amount of CDM and PHB concentration than those obtained from pBAD/Thio-TOPO-*phaCAB*_A–04_-expressing *E. coli* TOP10. Finally, glucose concentration was varied from 0, 2, 5, 10, 20, 50 g/L for both pBAD/Thio-TOPO-*phaCAB*_A–04_-expressing *E. coli* TOP10 and pBAD/Thio-TOPO-*phaCAB*_A–04_-expressing *E. coli* JM109. The optimal glucose concentration was 20 g/L for both recombinant strains, but pBAD/Thio-TOPO-*phaCAB*_A–04_-expressing *E. coli* TOP10 offered higher CDM (g/L), PHB (g/L), Y_*P/S*_ (g-PHB/g-glucose), and PHB productivity (g/L⋅h) than those obtained from *E. coli* JM109 as shown in Table 2.

**TABLE 2:** Comparison of kinetic parameters obtained from flask cultivation and fed-batch cultivation for PHB production from glucose between pBAD/Thio-TOPO-*phaCAB*_A–04_ and pColdTF-*phaCAB*_A–04_ expressing *E. coli* strains JM109, TOP10, and XL1-Blue in LB medium and MR medium.

**Kinetic parameters**	**pBAD/Thio-TOPO-*phaCAB*_A–04_**		**pBAD/Thio-TOPO-*phaCAB*_A–04_**		**pColdTF-*phaCAB*_A–04_**	
	**Flask**	**Flask**	**Fermentor**	**Fermentor**	**Fermentor**	**Fermentor**
Host strain	JM109	TOP10	XL1-Blue	XL1-Blue	XL1-Blue	XL1-Blue
Medium	LB	LB	MR	LB	LB	MR
Mode of operation	Batch	Batch	Fed-batch	Fed-batch	Fed-batch	Fed-batch
Glucose (g/L)	20	20	248	352	189	206
Maximum PHB concentration (g/L)	2.6 ± 0.2	5.6 ± 0.4	15.5 ± 0.5	29.0 ± 1.1	7.9 ± 0.6	31.4 ± 0.9
Maximum CDM (g/L)	3.9 ± 0.8	6.1 ± 1.1	46.7 ± 0.8	51.2 ± 1.0	28.2 ± 0.9	39.3 ± 1.2
% Maximum PHB content (w/w)	66.7 ± 2.2	93.3 ± 0.9	29.1 ± 1.8	60.2 ± 2.3	28.0 ± 1.5	83.0 ± 3.8
Specific growth rate (1/h)	0.08	0.02	0.06	0.04	0.06	0.05
Specific consumption rate (g-glucose/g-CDM/h)	0.31	0.38	0.38	0.21	0.11	0.22
Specific production rate (g-PHB/g-CDM/h)	0.02	0.12	0.06	0.09	0.01	0.09
Y_*X/S*_ (g-CDM/g-glucose)	0.03	0.004	0.34	0.11	0.3	0.11
Y_*P/S*_ (g-PHB/g-glucose)	0.1	0.28	0.16	0.21	0.10	0.39
Productivity (g/(L.h)	0.1	0.2	0.26	0.4	0.2	0.6
Time (h)	24	24	58	72	54	54

Transmission electron microscopy analysis (Figure 5B) revealed the morphology of PHB granules accumulating in pBAD/Thio-TOPO-*phaCAB*_A–04_-expressing *E. coli* TOP10. Most recombinant cells contained only one single PHB granule varying in size. Finally, PHB was extracted from cells, purified and subjected to chemical structure analysis by 2D-^1^H NMR and thermal analysis by DSC. The chromatogram of 2D-^1^H NMR when compared with the PHA chemical structure in previous reports showed that the homopolymer PHB was produced (data not shown). The melting temperature, T_*m*_, of all the PHB film samples produced in this study was in the range of 172–176°C (Owen et al., 1992), and the glass transition temperature, T_*g*_, was in the normal range of 0–1.5°C (Shimamura et al., 1994; Doi et al., 1995; Chanprateep et al., 2010). Altogether, under shaken flask experiments, the optimal conditions consisting of a low inoculum size of 4.5 × 10^8^ CFU/mL and LB medium containing 50 μg/ml carbenicillin supplemented with 1% (w/v) L-arabinose yielded 6.1 ± 1.1 g/L cell dry weight, a PHB content of 93.3 ± 0.9% (w/w) and a productivity of 0.2 g/(L⋅h), whereas the wild-type *C. necator* strain A-04 accumulated 78% (w/w) PHB with a productivity of 0.09 g/(L⋅h).

### Comparison of PHB Production Between pBAD/Thio-TOPO-*phaCAB*_A–04_ and pColdTF-*phaCAB*_A–04_ in a 5-L Fermenter by the pH-Stat Strategy

However, to attain high cell density cultivation, *E. coli* TOP10 was not a good candidate because of the limitation to attain high cell density in fed-batch cultivation experiments (data not shown). As discussed with a well-known expert in the field, *E. coli* XL1-blue is highly recommended to be used as a host strain (personal communication with Professor Takeharu Tsuge at Tokyo Institute of Technology). In addition, the optimal conditions for *phaCAB*_H16_ expression by arabinose P_BAD_ promoter in *E. coli* XL1-blue have already been reported and were the same conditions as in our study. Thus, in fed-batch cultivation experiments, we changed the host strain from *E. coli* TOP10 to *E. coli* XL1-blue and compared its potential PHB production efficiency between pBAD/Thio-TOPO-*phaCAB*_A–04_ and pColdTF-*phaCAB*_A–04_ in fed-batch cultivation experiments by applying the pH-stat strategy that was previously reported by Kahar et al. (2005) with some modifications by comparing LB medium and MR medium. The objective of this study was to compare araBAD promoter (pBAD/Thio-TOPO, arabinose induction) with the cspA promoter (pColdTF, cold temperature and IPTG induction) because there is no data supporting the use of these promoters when expressing *phaCAB* in high cell density of recombinant *E. coli* in fed-batch cultivation. In addition, our research group have studied and compared pColdTF-*phaCAB*_A–04_ with pGEX-6P-1, pColdI, pColdTF, pBAD/Thio-TOPO, and pUC19 (native promoter) in *E. coli* JM109 and found that pColdTF was the best among various promotors tested in shake flask cultivations (manuscript submitted for publication). Therefore, pColdTF-*phaCAB*_A–04_ was chosen in this study and compared with pBAD/Thio-TOPO-*phaCAB*_A–04_ using the same host strain.

The fed-batch culture with pH-stat feeding of glucose was carried out using *E. coli* XL1-blue and compared between pBAD/Thio-TOPO-*phaCAB*_A–04_ and pColdTF-*phaCAB*_A–04_. In addition, MR medium (Kahar et al., 2005), which is a synthetic medium, was also compared with LB medium, which is a complex medium. For pBAD/Thio-TOPO-*phaCAB*_A–04_ expressing *E. coli* XL1-blue in LB and MR medium, the culture conditions were controlled for optimal cell growth and PHB production as obtained from the above experiments. At 24 h, expression of the *pha* operon was induced by the addition of IPTG to a final concentration of 0.5 mM to promote the expression of *phaCAB*_A–04_ and obtain PHB production. For comparison, pColdTF-*phaCAB*_A–04_ expressing *E. coli* XL1-blue in LB and MR medium was performed in the same manner.

**FIGURE 6:**
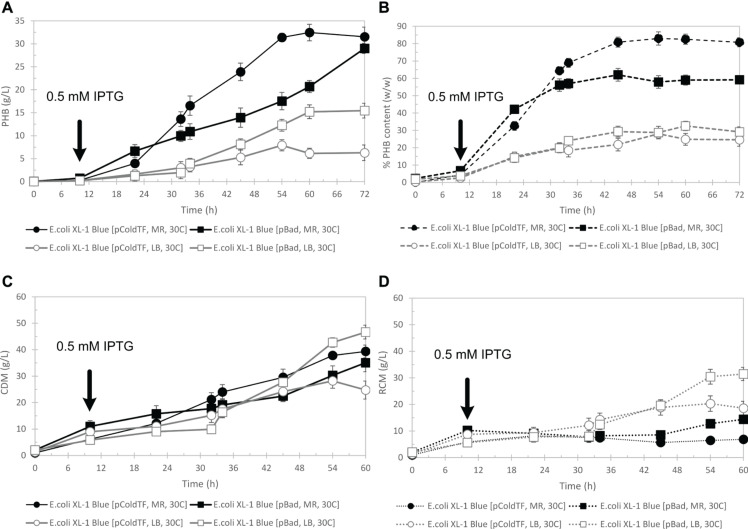
Time courses of PHB g/L **(A)**, PHB content % (w/w) **(B)**, CDM (g/L) **(C)**, and RCM (g/L) **(D)** during batch cultivation in a 5-L fermenter in comparison between pBAD/Thio-TOPO-*phaCAB*_A–04_ and pColdTF-*phaCAB*_A–04_-expressing *E. coli* XL1 blue-cultured in LB medium and MR medium.

Figure 6 shows the time courses of CDM (g/L) (Figure 6C), PHB (g/L) (Figure 6A), and PHB content (% w/w) (Figure 6B) during batch cultivation in a 5-L fermenter, comparing pBAD/Thio-TOPO-*phaCAB*_A–04_ and pColdTF-*phaCAB*_A–04_ expressing *E. coli* XL1-blue when cultivated in LB medium and MR medium. After 54 h, the CDM of 39.3 ± 1.2 g/L and PHB 31.4 ± 0.9 g/L with 83.0 ± 3.8% (w/w) of PHB content were obtained from pColdTF-*phaCAB*_A–04_ expressing *E. coli* XL1-blue in MR medium whereas CDM of 51.2 ± 1.0 g/L and PHB 29.0 ± 1.1 g/L with 60.2 ± 2.3% (w/w) of PHB content were obtained from pBAD/Thio-TOPO-*phaCAB*_A–04_ expressing *E. coli* XL1-blue in MR medium. In MR medium, the PHB yield from glucose, Y_*P/S*_, was 0.21 for pBAD/Thio-TOPO-*phaCAB*_A–04_ and 0.39 for pColdTF-*phaCAB*_A–04_. However, in LB medium, Y_*P/S*_ was 0.16 for *E. coli* XL1-blue harboring pBAD/Thio-TOPO-*phaCAB*_A–04_ and 0.10 for *E. coli* XL1-blue harboring pColdTF-*phaCAB*_A–04_ (Table 2). The highest productivity (PHB in g/L⋅h) was 1.9 g-PHB/L⋅h for pColdTF-*phaCAB*_A–04_, which was slightly higher than the 1.4 g-PHB/L⋅h of pBAD/Thio-TOPO-*phaCAB*_A–04_ expressing *E. coli* XL1-blue.

## Discussion

*Cupriavidus necator* strain A-04 was demonstrated in previous studies to synthesize various types of monomeric PHAs from a variety of carbon sources. It was morphologically and taxonomically identified, and 16S rRNA gene analysis revealed that the isolate was close to *C. necator* H16 with different cell morphologies, PHA yields, productivity and growth rates on the same carbon source. To date, a number of transcription-based inducible systems have been tested in *C. necator* H16 (Bhubalan et al., 2008; Fukui et al., 2011), and a number of recombinant *phaCAB*_H16_-expressing *E. coli* have been constructed (Kahar et al., 2005; Rehm and Steinbüchel, 2005; Agus et al., 2006). Therefore, we would like to challenge the expression of *phaCAB*_A–04_ in *E. coli* and investigate the optimal conditions that promoted high cell density with high PHB production. Among various types of promoters, there have been few reports on the application of arabinose-inducible expression systems (Fukui et al., 2011). Thus, in this study, *phaCAB*_A–04_ was cloned and expressed in pBAD/Thio-TOPO (araBAD promoter, arabinose-inducible vector, N-terminal thioredoxin fusion protein and C-terminal 6His-fusion protein). First, four different media (LB, 2YT, SB, and TB media) were examined for PHB production in shake flask experiments. It was found that TB medium promoted the highest cell formation because it contained the highest amount of yeast extract (rich nitrogen source), but not suitable for promoting PHB production. In contrast, LB medium contained the lowest amount of yeast extract and tryptone with a slightly higher amount of sodium chloride and was found to promote PHB production better than TB, 2YT and SB media. This finding was in accordance with Wang et al. (2009) who investigated various amount of nitrogen (NH_4_Cl, 0.2 g/L, 1 g/L, 2.5 g/L, 5 g/L) in M9 medium supplied with glucose as carbon source and who concluded that low nitrogen concentration led to the fast and high PHB accumulation in their engineered *E. coli*. The more NH_4_Cl supplied in the medium, the less PHB accumulated in the cells that could support our results. From Figure 3B, % PHB content (w/w) went down for LB medium over time after 12 h whereas RCM remained constant and PHB production slightly decreased after 30 h. This phenomenon caused by the decrease of organic nitrogen source and Mg^2+^ in LB medium was not sufficient, which were discussed again in fed-batch cultivation as feeding of Mg^2+^ was performed in both LB medium and MR medium to improve this limitation (Nikaido, 2009). Next, basically, the expression level via the araBAD promoter could be tightly regulated by varying the arabinose concentration. We found that 1% L-arabinose was the optimum concentration, which was the same concentration that was previously reported (Horng et al., 2010). Horng et al. (2010) reported co-expression of *phaCAB*_H16_ and *vgb* (bacterial hemoglobin from *Vitreoscilla stercoraria*) constructed and controlled by the same arabinose P_BAD_ promoter in *E. coli* XL1-blue. The optimal arabinose concentration for maximum inductions of pha*CAB*_H16_ was 1%, and there was no significant increase in PHB production at more than 2% arabinose, which was similar to our finding. In shaken flask experiments, pBAD/Thio-TOPO-*phaCAB*_H16_ and *vgb* expressing *E. coli* XL1-blue, CDM 5.13 ± 0.02 g/L with 3.06 ± 0.02 g/L (59.51 ± 0.17% w/w) were obtained which was slightly lower than our results, pBAD/Thio-TOPO-*phaCAB*_A–04_ expressing *E. coli* TOP10, with 6.1 ± 1.1 g/L of CDM, PHB content of 93.3 ± 0.9% (w/w) and productivity of 0.2 g/(L⋅h). The reason may be from some differences, including the use of a low inoculum size of 4.5 × 10^8^ CFU/mL and LB medium containing 2% glucose and 50 μg/ml carbenicillin at 37°C, while Horng et al. (2010) used LB medium containing 1% glycerol and 65 μg/ml ampicillin at 37°C. Nevertheless, Wu et al. (2016) also reported the use of the L-arabinose promoter to overexpress tubulin-like protein (*FtsZ*) and MinC, MinD and MinE, which regulate the formation of division sites. The optimal L-arabinose concentration was reported to be 0.2% for plasmid p15a-Pbad-ftsQLWN in *E. coli* JM109Δ*minCD*, resulting in 11.58 g/L CDM with a PHB content of 82.13% (w/w) in LB medium containing 20 g/L glucose at 30°C. The decrease of PHB production with 2% L-arabinose induction can be referred to the study of Khlebnikov et al. (2000) who reported that genes encoding arabinose catabolic genes (*araBAD*) are under arabinose-inducible control through AraC; arabinose binds to the AraC protein, which positively regulates the expression of transporters and negatively regulates its own expression. They investigated the effects of the arabinose concentration and arabinose-independent transport control on population homogeneity using flow cytometry. It was reported that arabinose concentration of 2% was a saturate concentration and a decline in the specific fluorescence was observed. This phenomenon might be ascribed to arabinose catabolism, resulting in a lower internal arabinose concentration (Khlebnikov et al., 2000) and lower PHB production in our case. The morphology of pBAD/Thio-TOPO-*phaCAB*_A–04_-expressing *E. coli* TOP10 containing PHB granules was observed by TEM, and most cells contained a single PHB granule varying in size.

In fed-batch experiments, a pH stat strategy employing ammonium hydroxide solution to maintain glucose concentration (Kahar et al., 2005) was applied in this study, and it was found that the pH stat strategy effectively worked well for both pBAD/Thio-TOPO-*phaCAB*_A–04_ expressing *E. coli* XL1-blue and pColdTF-*phaCAB*_A–04_-expressing *E. coli* XL1-blue in LB medium and MR medium. The pH-stat strategy together with MR medium promoted PHB production by both pBAD/Thio-TOPO-*phaCAB*_A–04_ expressing *E. coli* XL1-blue (CDM of 51.2 ± 1.0 g/L and PHB 29.0 ± 1.1 g/L with 60.2 ± 2.3% (w/w) PHB content) and pColdTF-*phaCAB*_A–04_ expressing *E. coli* XL1-blue (CDM of 39.3 ± 1.2 g/L and PHB 31.4 ± 0.9 g/L with 83.0 ± 3.8% (w/w) PHB content). We found that LB medium promoted higher cell density than that obtained from MR medium, but LB medium resulted in lower PHB production than MR medium. Figures 6C,D shows that pBAD/Thio-TOPO-*phaCAB*_A–04_-expressing *E. coli* XL1-blue grown in LB medium attained a CDM of 46.7 ± 0.8 g/L and PHB 15.5 ± 0.5 g/L with 29.1 ± 1.8% (w/w) PHB content. The low content of PHB may have resulted from the LB medium containing insufficient mineral compounds to promote PHB biosynthesis. In addition, MR medium contained a high concentration of Mg^2+^, in which *E. coli* required high amounts of Mg^2+^ and Ca^2+^ to bridge the highly negatively charged lipopolysaccharide (LPS) molecules in its outer membrane. The total amount of Mg^2+^ associated with *E. coli* cells is estimated to be approximately 100 mM, where LB broth contains only between 30 and 200 μM Mg^2+^, so that the amount of Mg^2+^ in LB is not sufficient (Nikaido, 2009). During cultivation in LB medium, *E. coli* cells undergo alterations in carbon nutrition. Cells subsequently use easier-to-utilize amino acids until they are depleted and then switch to harder-to-use amino acids (Sezonov et al., 2007), resulting in a slow specific production rate and growth rate. During cultivation in MR medium, *E. coli* cells growing in glucose-minimal medium convert glucose into acetate and other organic acids during the early exponential phase; then, in the mid-exponential phase, they grow primarily by oxidizing these organic acids. It has been reported that MgATP is the substrate of numerous phosphorylating enzymes and the principal energy source of the cell. Indeed, any increase in metabolic activity increases the rate of MgATP use and, consequently, the rate of ADP and magnesium release, and vice versa (Gout et al., 2014). Therefore, because MR medium and the feeding solution contained a greater amount of Mg^2+^ than those in LB medium, they enhanced the metabolic advantage of *E. coli* cells cultured in this medium as well as supplemented Mg^2+^ together with glucose using fed-batch cultivation techniques. However, the same composition of glucose, MgSO_4_⋅7H_2_O and thiamine in the feeding solution was used for both MR medium and LB medium. We found that LB medium together with Mg^2+^ feeding promoted only cell growth but did not enhance PHB biosynthesis. Gahlawat and Srivastava (2012) studied the optimum concentration of medium components obtained from statistical design to identify the batch growth and product kinetics parameters of PHB fermentation in *Azohydromonas australica* using sucrose. They reported that mineral (essential) nutrients (MgSO_4_⋅7H_2_O, KH_2_PO_4_, and Na_2_HPO_4_), which were similar to MR medium (nutrients (MgSO_4_⋅7H_2_O, KH_2_PO_4_, and (NH_4_)_2_PO_4_), play a very important role in the susceptibility of the buffering capacity of the nutrient medium, which is necessary to support the growth of microbial cells and PHB production.

## Conclusion

This study demonstrates that the araBAD promoter and thioredoxin fusion proteins are able to yield high cell density and PHB production in fed-batch cultivation using a pH-stat feeding strategy. To achieve economical productivity, we found that MR medium with a pH stat feeding strategy (NH_4_OH) coupled with a sufficient amount of Mg^2+^ in glucose feeding solution is an important key to attaining both a high cell density of CDM and PHB production. LB medium plus Mg^2+^ in glucose feeding solution promoted only high cell density. However, the advantage of the P_BAD_ expression system is that the cost of arabinose is cheaper than that of the IPTG induction system. In this study, we compared araBAD promoter (pBAD/Thio-TOPO, arabinose induction) with the cspA promoter (pColdTF, cold temperature and IPTG induction) and attained high cell density in both cases. Therefore, pBAD/Thio-TOPO-*phaCAB*_A–04_ expressing *E. coli* XL1-blue and pColdTF-*phaCAB*_A–04_ expressing *E. coli* XL1-blue constructed by our research group possess a high potential for PHB production.

## Data Availability Statement

The datasets presented in this study can be found in online repositories. The names of the repository/repositories and accession number(s) can be found below: https://www.ncbi.nlm.nih.gov/genbank/, FJ897463.1; https://www.ncbi.nlm.nih.gov/genbank/, FJ897461.1; and https://www.ncbi.nlm.nih.gov/genbank/, FJ897462.1.

## Author Contributions

SCN and SV performed the experiments. OP provided guidance and suggestions for the experimental design and discussed the results. KH and KO provided guidance and suggestions. SCN provided guidance and suggestions for the experimental design, discussed the results, and wrote and edited the manuscript. All authors read and approved the final version of the manuscript.

## Conflict of Interest

The authors declare that the research was conducted in the absence of any commercial or financial relationships that could be construed as a potential conflict of interest.

## Supplementary Material

Supplementary Figure 1 Growth profile on various glycerol concentrations of *C. necator* strain A-04 (green line), *C. necator* H16 (red line) and variant V6C6 (blue line) that is an adaptive laboratory evolution of *C. necator* H16. This experiment was performed by Dr. Tuck Seng Wong (University of Sheffield, United Kingdom) under the research collaboration between National Center for Genetic Engineering and Biotechnology (BIOTEC) and Chulalongkorn University.
