# Supplementary figures and images for: Polyhydroxybutyrate (PHB) Production Using an Arabinose-Inducible Expression System in Comparison With Cold Shock Inducible Expression System in Escherichia coli
Source: Front Bioeng Biotechnol. 2021 May 3;9:661096. doi: 10.3389/fbioe.2021.661096 (PMC8126650; doi:10.3389/fbioe.2021.661096)

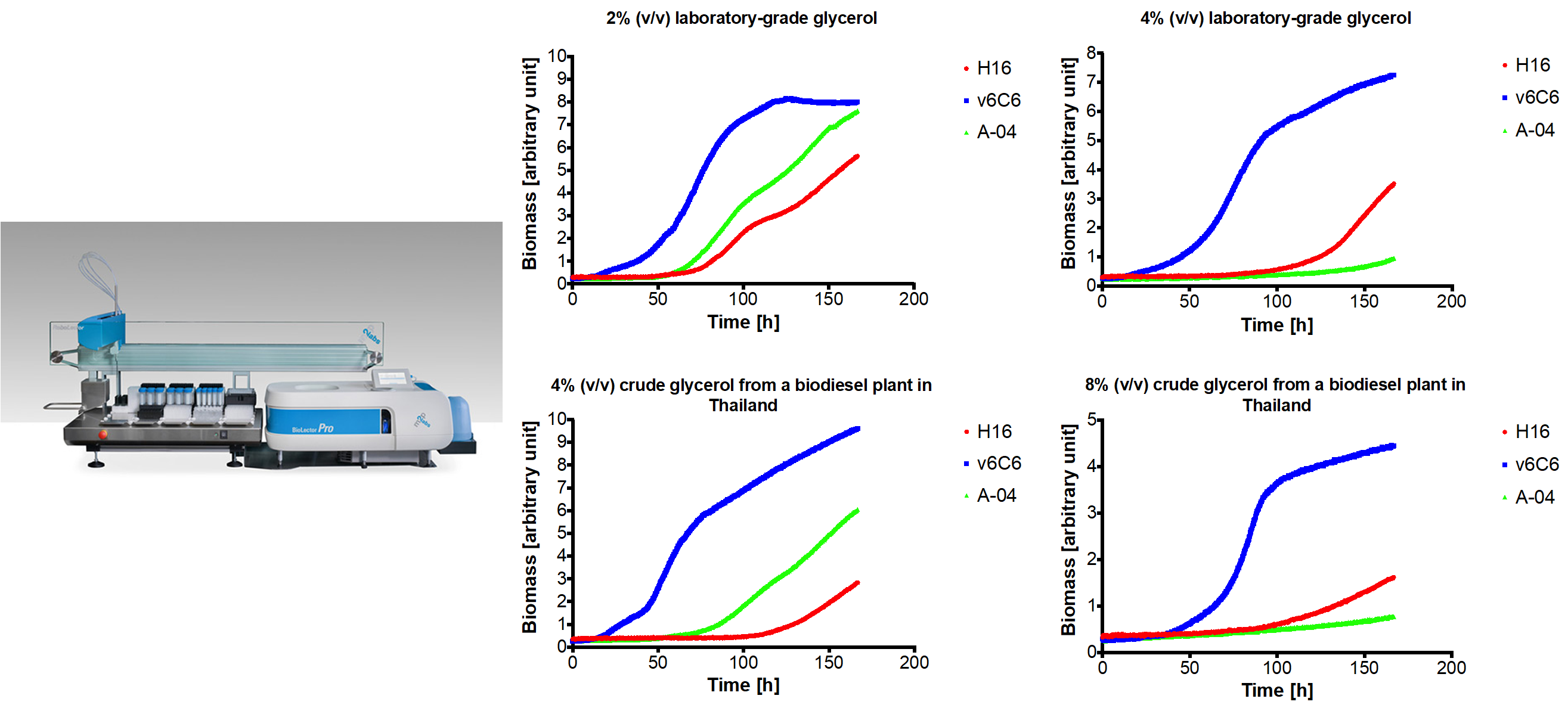

Supplement: Supplementary Figure 1 — Growth profile on various glycerol concentrations of C. necator strain A-04 (green line), C. necator H16 (red line) and variant V6C6 (blue line) that is an adaptive laboratory evolution of C. necator H16. This experiment was performed by Dr. Tuck Seng Wong (University of Sheffield, United Kingdom) under the research collaboration between National Center for Genetic Engineering and Biotechnology (BIOTEC) and Chulalongkorn University. [file Image_1.tif]

## Slide 1
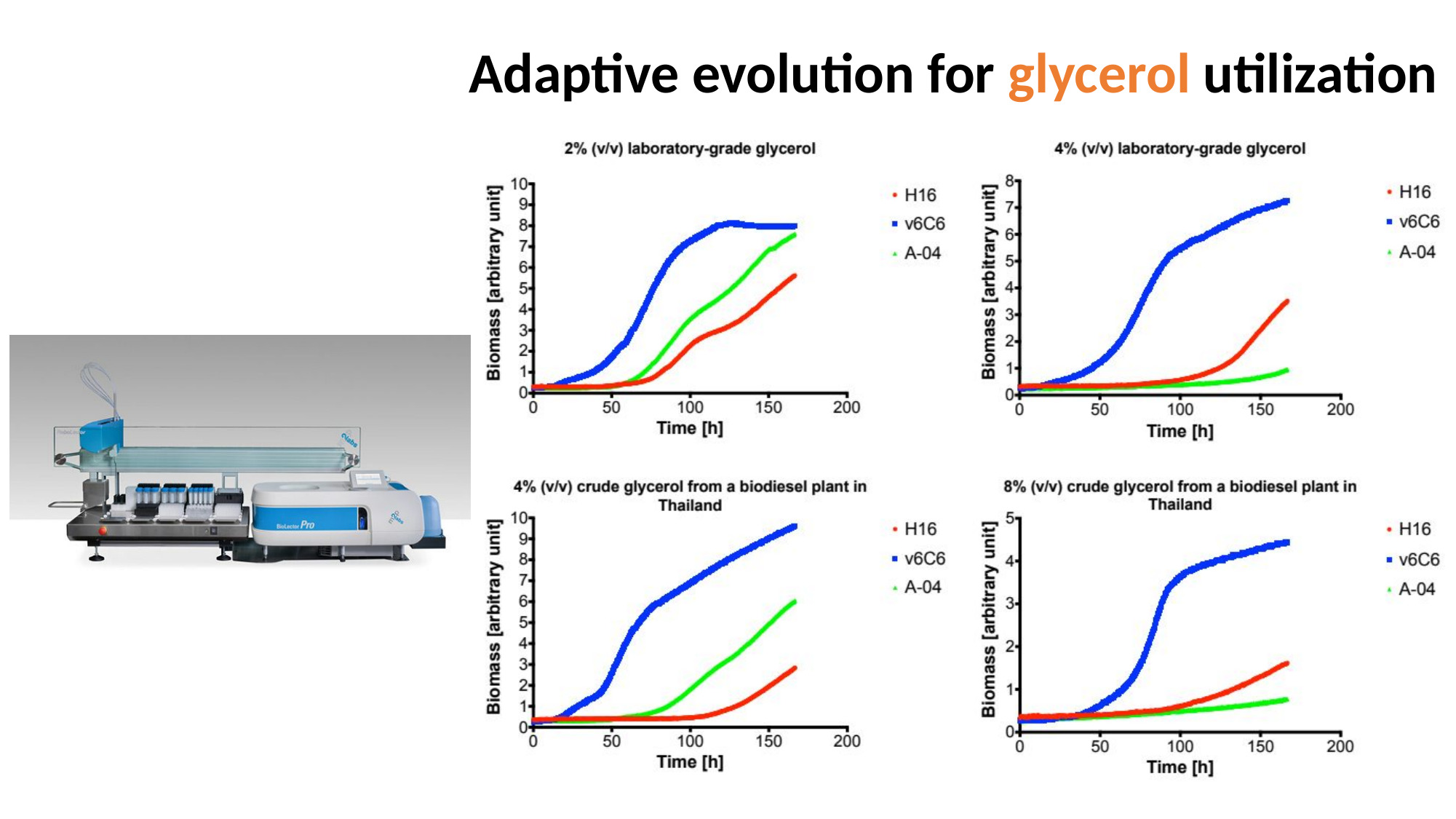

Adaptive evolution for glycerol utilization

Supplement: Supplementary file 2 [file Presentation_1.PPTX]
